# Physicians’ Visiting List for ’87

**Published:** 1887-01

**Authors:** 


PHYSICIAN' VISITING LIST FOR 1887.—P. Blakiston, Son & Co., the old and enterprising publishers are the first in the field with the "List" for 1887, the 36th year of its publication. As usual, it is up to the latest requiremts, each succeding number showing some improvement. This one is gotten up with reference to convenience; is printed on excellent paper, with clear new type, contains a pocket, and nickle-headed pencil; is bound in a compact substantial form, and contains blanks for a number of purposes and the usual tables of doses, incompatibles, etc. Price, $1.00, larger size, $1.25.

Mention this JOURNAL, please.

